# Development of a novel immunoproteasome digestion assay for synthetic long peptide vaccine design

**DOI:** 10.1371/journal.pone.0199249

**Published:** 2018-07-03

**Authors:** Hiroshi Wada, Atsushi Shimizu, Toshihiro Osada, Yuki Tanaka, Satoshi Fukaya, Eiji Sasaki

**Affiliations:** Discovery and Preclinical Research Division, Taiho Pharmaceutical Co. Ltd., Tsukuba, Ibaraki, Japan; Monash University, AUSTRALIA

## Abstract

Recently, many autologous tumor antigens have been examined for their potential use in cancer immunotherapy. However, the success of cancer vaccines in clinical trials has been limited, partly because of the limitations of using single, short peptides in most attempts. With this in mind, we aimed to develop multivalent synthetic long peptide (SLP) vaccines containing multiple cytotoxic T-lymphocyte (CTL) epitopes. However, to confirm whether a multivalent vaccine can induce an individual epitope-specific CTL, the only viable screening strategies currently available are interferon-gamma (IFN-μ enzyme-linked immunospot (ELISPOT) assays using human peripheral blood mononuclear cells, or expensive human leukocyte antigen (HLA)-expressing mice. In this report, we evaluated the use of our developed murine-20S immunoproteasome (i20S) digestion assay, and found that it could predict the results of IFN-μ ELISPOT assays. Importantly, the murine-i20S digestion assay not only predicted CTL induction, but also antitumor activity in an HLA-expressing mouse model. We conclude that the murine-i20S digestion assay is an extremely useful tool for the development of “all functional” multivalent SLP vaccines.

## Introduction

Cancer immunotherapy represents a major breakthrough in cancer treatment with the emergence of immune-checkpoint inhibitors such as anti- programmed death 1 (PD-1) and anti- cytotoxic T lymphocyte antigen 4 (CTLA4) [1, 2]. In immunotherapeutic approaches, cancer vaccines have also been expected to elicit antitumor responses through the activation of the immune system, and become a novel therapeutic technique.

Peptides are considered good drug candidates due to their unique advantages of low molecular weight, high selectivity, and low toxicity in normal tissues [3]. Over the last 20 years, many human tumor-associated antigens (TAAs) have been identified, and clinical studies of epitope peptides derived from the sequence of TAAs have been carried out in patients with metastatic cancer. These peptide-based cancer vaccines activate cytotoxic T-lymphocytes (CTLs) which recognize the identical antigenic peptides presented on the surface of cancer cells. However, almost all clinical studies of cancer peptide vaccines have failed to demonstrate clinical benefit [4]. Major vaccination approaches have included the use of single, short peptides of TAA epitope sequences [5–7]. However, it has been pointed out that vaccination with short peptides is far from optimal because immunological tolerance can be induced [8, 9]. Therefore, in order to induce effective T-cells reactive against as many target antigens as possible, several alternative strategies have been investigated, including peptide cocktail vaccines [10], multi-epitope vaccines containing CTL-epitopes and helper-epitopes [11], personalized peptide vaccines [12], and multivalent synthetic long peptide (SLP) vaccines [13, 14].

With respect to multivalent long peptide vaccines, prophylactic vaccines for human papillomavirus have already been approved for clinical use. With the inclusion of nine different epitopes within the long peptide, one of them is expected to cover approximately 90% of cervical cancers [15, 16]. In addition, elongation of epitope presentation and strong CTL induction has been reported using long peptides [17–19], compared to the down-modulation of tolerance via dendritic cell (DC)-selective antigen incorporation and presentation seen with shorter epitope peptides [20, 21]. Based on the available information, multivalent SLPs that consist of several CTL epitopes may be necessary for the development of cancer peptide vaccines suitable for wide patient coverage.

The induction of epitope-specific CTLs in multivalent SLP vaccines is related to the position of each epitope and their optimum orientation, which need to be decided empirically [22]. However, there are screening strategies that can be used to confirm whether multivalent SLP vaccines can induce all individual epitope-specific CTLs. Though in vivo screening using mice expressing human leukocyte antigen (HLA) molecules has been reported, such studies are expensive and the procedure is complicated [23]. Therefore, a simpler screening method is greatly in demand.

In this report, we describe a new screening method, the murine-i20S digestion assay, for selecting highly potent multivalent SLP vaccines using the 20S immunoproteasome (i20S). The i20S is a multi-subunit protease and its active site is composed of three catalytic β-subunits: β1i (LMP2), β2i (MECL-1), and β5i (LMP7) [24, 25]. These i20S-specific subunits are induced by interferon-gamma (IFN-γ), and the resulting i20S differs from the standard proteasome in regard to proteolytic activity [26–28]. During epitope cleavage by DCs, the i20S firstly generates an N-extended version of the antigenic peptide [29, 30]. We evaluated whether the murine-i20S digestion assay can predict not only CTL induction, but also HLA-dependent antitumor effects in a mouse model. Based on the results, we conclude that the murine-i20S digestion assay can be used to select efficacious multivalent SLP vaccines.

## Materials and methods

### Reagents

Fmoc-amino acid, Fmoc-amino acid alko-PEG resin, 2-(1H-benzotriazole-1-yl)–1,1,3,3-tetramethyl-uronium hexafluorophosphate (HBTU), 1-hydroxybenzotriazol (HOBt), and N,N-diisopropylethylamine (DIEA) were purchased from Watanabe Chemical Industries, Ltd (Hiroshima, Japan). N-methylpyrrolidone (NMP), methanol, methyl tert-butyl ether (MTBE), trifluoroacetic acid (TFA), thioanisole, ethanedithiol (EDT), thiophenol, 2-methylindole and acetonitrile were purchased from Nacalai Tesque Inc., (Kyoto, Japan). Ethyl methyl sulfide (EMS) was purchased from Sigma Aldrich (St. Louis, MO, USA).

### Peptide synthesis

All trivalent SLPs were synthesized on a Prelude peptide synthesizer (Protein Technologies, Inc., Tucson, AZ, USA) at a 40-μmol scale using standard Fmoc protocols. All amino acids were double coupled using 3-fold excess of Fmoc-amino acids relative to the Fmoc-amino acid alko-PEG resin (0.20–0.25 mmol/g, Watanabe Chemical, Hiroshima Japan). Fmoc deprotection was performed using 20% piperidine/NMP. Coupling was carried out using amino acid/HBTU/HOBt/DIEA (1:1:1:2) in NMP. NMP top washes (5 × 0.5 min) were performed between deprotection and coupling steps. One NMP top wash was used between the double couplings. After completion of couplings, one NMP top wash was performed before the next deprotection step. Cleavage was performed with TFA/H2O/thioanisole/EDT/EMS/thiophenol (82:5:5:3:2:3) plus 2-methylindole (10 mg/mL). Crude peptides were precipitated from cold ether and collected by centrifugation. The resulting crude peptides were purified with preparative reversed phase high performance liquid chromatography (HPLC) (Waters Corp., Manchester, UK) with an ODS column. All peptides were characterized using a Micromass ZQ mass spectrometer (Waters) equipped with an ESI source. Other peptides (epitope peptides, bivalent peptides) were synthesized and analyzed by Toray Research Center, Inc. (Tokyo, Japan).

### Mice

*HLA-A2* transgenic (Tg) mice were purchased from Taconic Biosciences, Inc. (Hudson, NY, USA). and *HLA-A24* and *HLA-A31* knock-in (KI) mice were generated in our group as previously described [23]. Mice were injected subcutaneously twice at intervals of 7 days with a mixture of peptides emulsified in incomplete Freund's adjuvant (IFA) or Montanide ISA-51VG (Seppic, Paris, France, 100 μg/100 μL/mouse). Seven days after the last immunization, mice were sacrificed and the lymph node cells or splenocytes were prepared. All animal procedures were conducted in compliance with National Institutes of Health guidelines and were approved by the Taiho Institutional Animal Care and Use Committee.

### Enzyme-linked immunospot (ELISPOT) assay

5×10^6^ cells in 1mL of RPMI-1640 containing 10% heat-inactivated fetal bovine serum (FBS), 100 U/mL Penicillin, 100 μg/mL Streptomycin, and 50 μM 2-Mercaptoethanol (ME) were seeded in a 24-well plate, and cultured for 8 days with target peptide (10 μg/mL), mouse recombinant interleukin (rIL)-15 (100 ng/mL) and mouse rIL-21 (100 ng/mL) [31]. After 8 days, living cells collected by density gradient centrifugation using Lympholyte-M (Cedarlane, Hornby, ON, Canada) were harvested and 2 × 10^5^ cells were cultured on anti-IFN-γ antibody coated plates (GEN-PROBE Inc., San Diego, CA, USA) with 2 × 10^5^ irradiated (30Gy) splenocytes and target epitope peptide (10 μg/mL), or negative control peptide (WT1p126 for HLA-A*0201; Her2p63 for HLA-A*2402; HIV-1 gp41_770-780_ for HLA-A*3101; all 10 μg/mL). After overnight incubation at 37°C, the IFN-γ producing cell spots were counted with an ELISPOT analyzer Immunospot S6 (Cellular Technology Ltd., Shaker Heights, OH, USA).

### i20S digestion

Both purified murine i20S and purified human i20S were purchased from Boston Biochem Inc. (Cambridge, MA, USA). 20 μg of SLP was dissolved in 300 μL of reaction buffer (20mM HEPES-KOH pH7.8, 2mM MgAc2, 2mM dithiothreitol) and incubated at 37°C with 2 μg of i20s. The reaction was stopped by adding 30 μL of acetic acid and mixing well with a vortex mixer, and then stored at −80°C until analysis. For the 0 h sample, acetic acid was pre-added before the reaction.

### Liquid chromatography-tandem mass spectrometry (LC-MS/MS) analysis

Digested peptides were subjected to analysis using a Synapt G2 HDMS (Waters Corp., Manchester, UK) equipped with an ACQUITY UPLC system (Waters Corp., Milford, MA, USA). Digested fragments were separated using an ACQUITY UPLC BEH C18 (1.7 μm, 2.1 mm × 100 mm; Waters Corp., Dublin, Ireland) using a mobile phase that contained solvent A (0.1% formic acid in water) and solvent B (0.1% formic acid in acetonitrile), at a flow rate of 400 μL/min. The mobile phase composition was held initially at 0% (v/v) solvent B, and then changed from 0% to 90% (v/v) solvent B over 60 min. The eluent was analyzed by MS/MS. The capillary voltage and cone voltage were set at 3 kV and 40 V, respectively, in the positive ion scan mode. A full mass spectrum was acquired over an m/z range of 100–6000, and the MSE technique was applied. Sequences of the digested fragments were assigned with the assistance of the BioLynx software (Waters). The identified cleavage peptides were aligned from the C-terminus, regardless of length, to draw digestion maps. If it was not possible to identify which amino-acids were included in a digested fragment, both amino acids were indicated using a white character.

### Tumor cells

The minigene of the squamous cell carcinoma antigen recognized by T-cells (SART2_93-101_), with an *HLA-A24* signal sequence, was cloned into the PiggyBac Dual Promoter vector along with a puromycin resistance cassette. The *HLA-A24* (HHD) fusion gene, consisting of human β2-microglobulin, *HLA-A24* (α1 and α2 domains) and the H-2 class I histocompatibility antigen D-B (*H-2D*^*b*^) (from the α3 domain to the C-terminus), was also cloned into a PiggyBac Dual Promoter vector (Transposagen Biopharmaceuticals, Inc., Lexington, KY, USA), this time with a hygromycin resistance cassette. These two vectors were then transfected into B16F10 murine melanoma cells obtained from the American Type Culture Collection (ATCC, Manassas, VA, USA). Subculture of the established cell line (named B16F10.A24/SART2_93-101_) was carried out every 3 days with Dulbecco’s Modified Eagle medium containing 10% heat inactivated FBS, 20 μg/mL puromycin dihydrochloride, and 500 μg/mL hygromycin B.

### Preventive vaccine model for SART2_93-101_-expressing syngeneic tumors

*HLA-A24* KI mice (n = 10) were subcutaneously injected with SART2_93-101_ SLP vaccine (30 nmol/mouse), or vehicle control, at the base of the tail. Administration was performed three times, at intervals of 7 days, and 7 days after the final vaccination, immunized mice were subcutaneously engrafted with B16F10.A24/SART2_93-101_ tumor cells (5 × 10^6^) into the right flank. Seven days after engraftment, tumor size was measured by caliper. Tumor volume was calculated using the formula: tumor volume (mm^3^) = length (mm) × [width (mm)]^2^/2

## Results

### Development of the murine-i20S digestion assay

**Fig 1:**
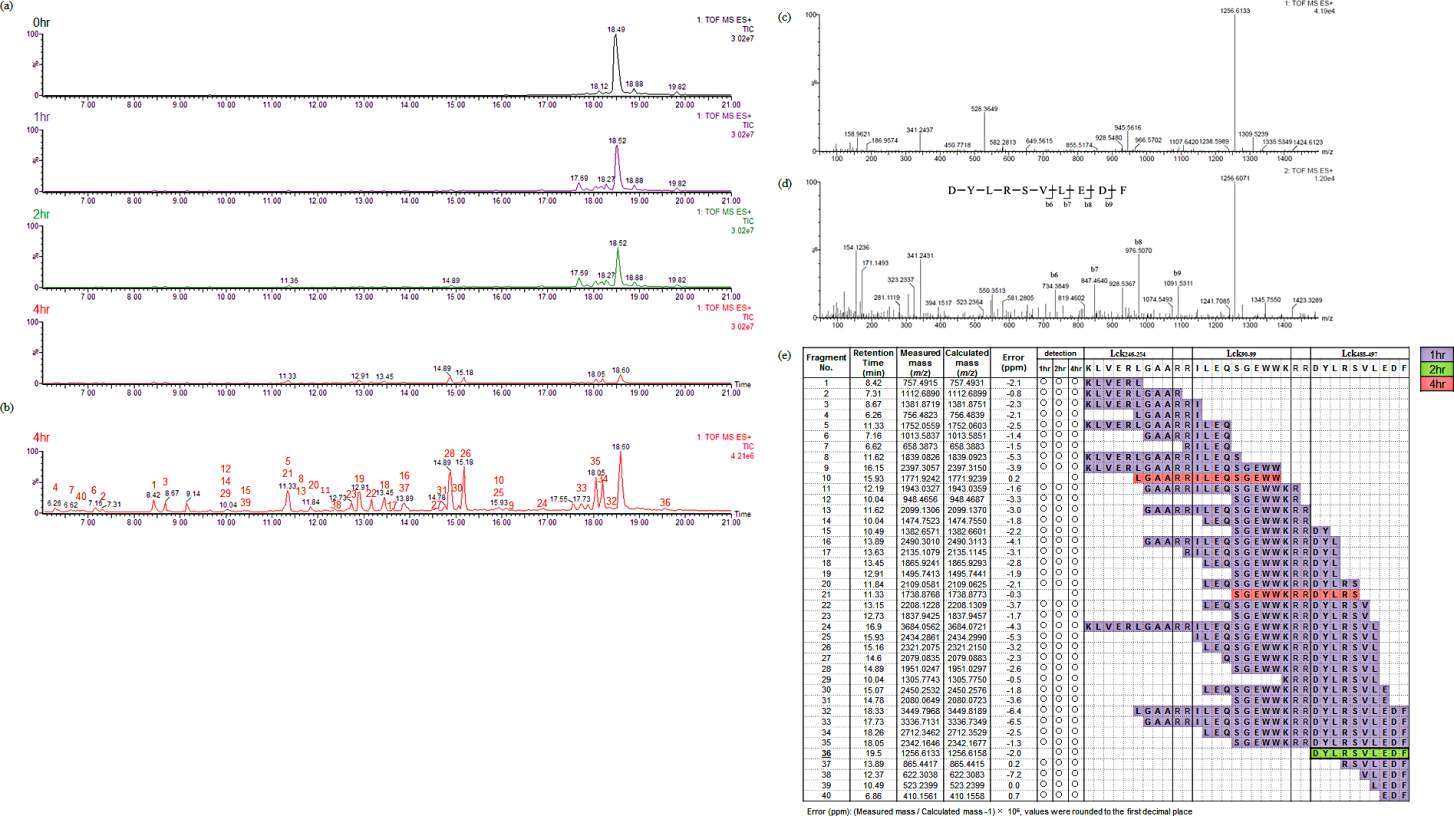
Time-dependent cleavage of trivalent SLP in the murine-i20S digestion assay. The i20S digested fragment was analyzed by MS/MS with a UPLC system. (a) Total ion chromatogram (TIC) traces of the bulk peptide products derived after 0, 1, 2, or 4 h digestion of the SLP (highlighted in black, purple, green, or red, respectively). (b) TIC shown in Fig 1A with change in the vertical axis scale (4 h time point) to optimize visualization of each peak. Numbers above each peak correspond to fragment numbers in Fig 1E. (c) Transformed MS spectrum of peak 36. The calculated mass of protonated DYLRSVLEDF is 1256.6157. (d) MS^E^ spectrum of peak 36. (e) “digestion map”. Each fragment is highlighted in purple, green, or red according to the first detected time point. The detected intact epitope is outlined in bold.

The murine-i20S digestion assay was developed using a trivalent SLP as a model peptide, which was composed of three human Lck-derived epitopes [32–34]. Including these three epitopes, epitopes used in this study are part of the group of peptides whose effects are indicated in clinical trials. Epitopes were linked with double arginine (Arg) residues to facilitate efficient cleavage of the individual epitopes, as well as to impart hydrophilicity [35, 36]. After the proteolytic reaction (either 1 h, 2 h or 4 h), the peptide-i20S mix was analyzed by LC. The time-dependent generation of neighbor peaks was observed near the original SLP peak (Fig 1A). Furthermore, many digested peptide fragments were identified (Fig 1B). The peptide sequence was determined as follows: First, LC-MS^E^ analysis was performed on the i20S digested sample by Q-TOF mass spectrometry. Both MS spectra and multiple MS/MS (MS^E^) spectra were obtained in one acquisition cycle. Second, the original MS spectra of each peak on TIC chromatograms which displayed doubly or triply charged ions were transformed to display the accurate monoisotopic mass, and peptide fragment with similar calculated masses in SLP were identified (Fig 1C). When the error ratio between measured mass and calculated mass was negligibly small (ppm < 10.0), the peptide sequence of the target fragment was used for identified. Third, for confirmation, fragment analyses were also performed using MS^E^ spectra (Fig 1D). The digested peptide fragment sequences were aligned to “digestion maps” as described in the methods, to make analysis easier (Fig 1E).

We decided to treat intact epitopes, and N-terminal extensions of one or two amino acids to intact epitopes (hereafter abbreviated as “epitope-related fragments”), as the analysis objects, since a previous report estimated that more than half (55–70%) of proteasome cleavage products have less than a two amino acid extension from the N-terminus [37]. For example, only the intact Lck_488-497_ epitope was detected as one of the epitope-related fragments after a 2 h-incubation sample in this result (Fig 1E). The incubation time-point is indicative of the ease with which fragments can be cleaved, however, the optimal incubation time for screening must be empirically determined.

### Validation of the murine-i20S digestion assay by comparison with CTL induction in HLA-expressing mice

Next, we performed a comparison of the results from the murine-i20S digestion assay and CTL induction, in order to validate the assay. Six trivalent SLPs, composed of three randomly-arranged human CTL epitopes [Wolf-Hirschhorn syndrome candidate 2 (WHSC2)_103-111_, W; SART3_302-310_, S3; and SART2_93-101_, S2] were synthesized (Table 1) [38–40]. The constructs containing these epitopes (W-S3-S2, S3-S2-W, S2-W-S3, S3-W-S2, W-S2-S3, and S2-S3-W) were then used in the murine-i20S digestion assay.

**Table 1:** Sequences of multivalent peptides.

Name	Orientation	Epitope Sequence
W-S3-S2	WHSC2_103-111_-RR-SART3_302-310_-RR-SART2_93-101_	**ASLDSDPWV**RR**LLQAEAPRL**RR**DYSARWNEI**
S3-S2-W	SART3_302-310_-RR-SART2_93-101_-RR-WHSC2_103-111_	**LLQAEAPRL**RR**DYSARWNEI**RR**ASLDSDPWV**
S2-W-S3	SART2_93-101_-RR-WHSC2_103-111_-RR-SART3_302-310_	**DYSARWNEI**RR**ASLDSDPWV**RR**LLQAEAPRL**
S3-W-S2	SART3_302-310_-RR-WHSC2_103-111_-RR-SART2_93-101_	**LLQAEAPRL**RR**ASLDSDPWV**RR**DYSARWNEI**
W-S2-S3	WHSC2_103-111_-RR-SART2_93-101_-RR-SART3_302-310_	**ASLDSDPWV**RR**DYSARWNEI**RR**LLQAEAPRL**
S2-S3-W	SART2_93-101_-RR-SART3_302-310_-RR-WHSC2_103-111_	**DYSARWNEI**RR**LLQAEAPRL**RR**ASLDSDPWV**
		*Epitope sequences are shown in bold*

**Fig 2:**
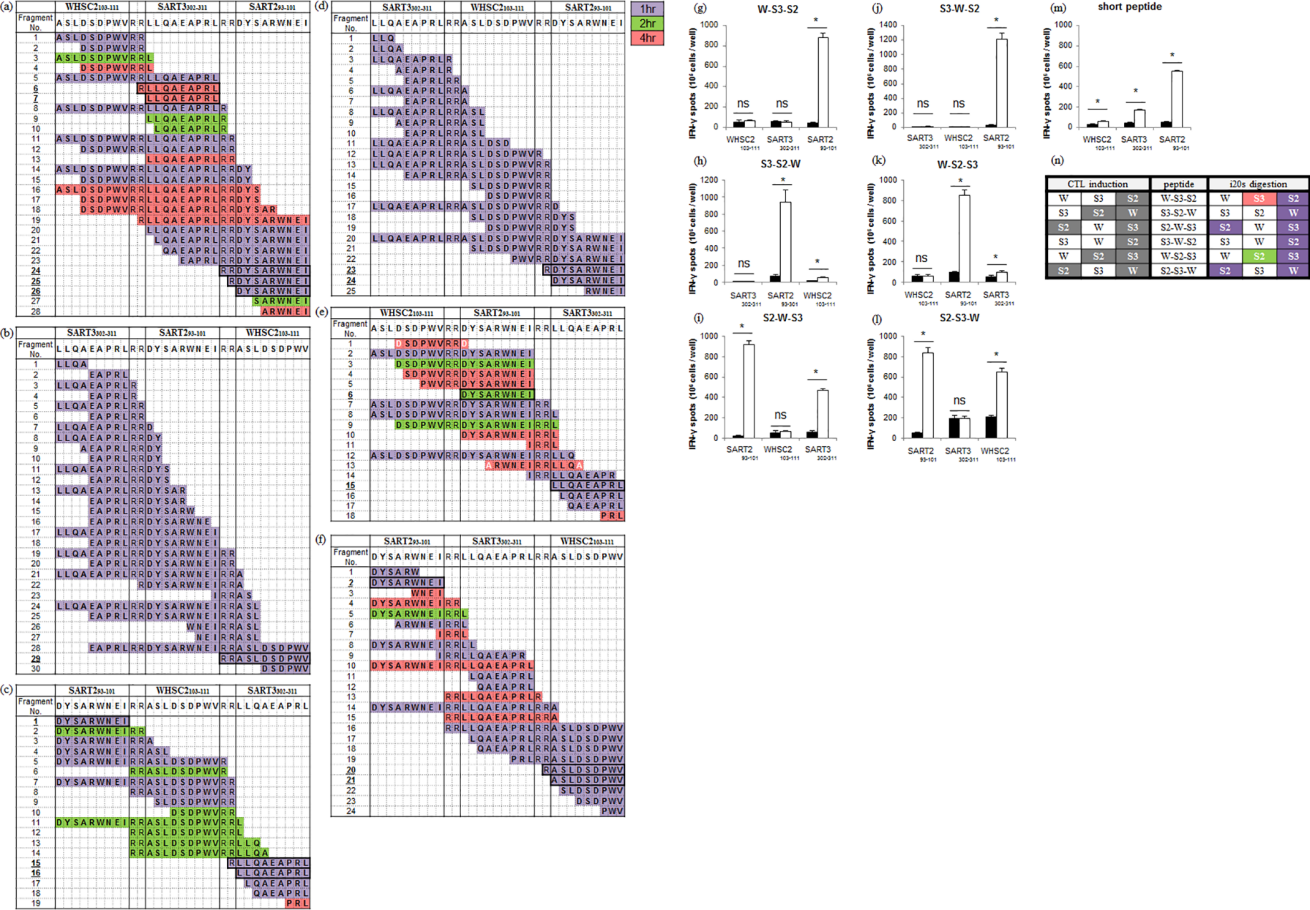
Comparison between the murine-i20S digestion assay and specific CTL induction using HLA-expressing mice. (a)-(f) The digestion maps of trivalent SLP fragments after 1, 2, or 4 h (highlighted in purple, green, or red, respectively). Epitope-related fragments are outlined in bold. (g)-(l) Results of IFN-γ ELISPOT in trivalent SLP-treated mice. Data represent mean ± s.d. (n = 3–4); open bars, target epitope peptide stimulation; closed bars, negative control peptide stimulation; * Student’s t-test, p < 0.05. (m) IFN-γ ELISPOT results in each short epitope peptide-treated mice. (n) Comparison of the results between CTL induction and the murine-i20S digestion assay. CTL-induced epitopes are colored in dark grey, and i20S digested epitopes are colored in purple, green or red, based on their first detected time-point i.e. 1, 2, or 4 h, respectively.

Epitope-related fragments of WHSC2_103-111_ and SART3_302-310_ were observed from the digested S3-S2-W, S2-S3-W constructs (Fig 2B & 2F) and W-S3-S2, S2-W-S3, W-S2-S3 constructs (Fig 2A and 2C & 2E), respectively. In the case of the SART2_93-101_, epitope-related fragments were detected from the five constructs except for the S3-S2-W (Fig 2A and 2C–2F). Although most of these epitope-related fragments were cleaved up to 1 h time point, the SART2_93-101_ epitope of W-S2-S3 was detected after the 2 h-incubation, and the epitope-related fragments of SART3_302-310_ in W-S3-S2 constructs were detected only from the 4 h sample.

Next, the actual CTL-inducing activity of six SLPs was confirmed using HLA-expressing mice. HLA-A*0201 transgenic (Tg) mice were used for WHSC2_103-111_ and SART3_302-310_, while HLA-A*2402 knock-in (KI) mice were used for SART2_93-101_, based on HLA-restrictions. The results showed that W-S3-S2 and S3-W-S2 induced specific CTLs only for SART2_93-101_ (Fig 2G & 2J). In contrast, S3-S2-W and S2-S3-W induced CTLs for WHSC2_103-111_ and SART2_93-101_ (Fig 2H & 2L), and S2-W-S3 and W-S2-S3 induced CTLs for SART3_302-310_ and SART2_93-101_ (Fig 2I & 2K). Compared to ELISPOT results for mice treated with short epitope peptides (Fig 2M), all SLPs were superior to short epitope peptides based on SART2_93-101_-specific spot numbers. In the case of WHSC2_103-111_ and SART3_302-310_, S3-S2-W, S2-S3-W, and S2-W-S3 were superior to short epitope peptides.

Fig 2N shows the comparison of the results between the murine-i20S digestion assay and the ELISPOT assay. The only false-positive result was the SART3_302-310_ epitope in W-S3-S2 after a 4 h-incubation, suggesting that a 4 h-incubation is not suitable for this assay. Antigen presentation has been reported to reach plateau levels at 3 h post-incubation [41]. Indeed, a 3 h-incubation of murine CD11c^+^ DCs with our multivalent SLP was able to activate specific CTLs in vitro (data not shown). Non-specific peptide cleavage might thus occur during longer incubations. Taking these observations together, we selected the 1 h and 2 h-incubation times as being the most suitable time points for screening assays. With the exception of the 4 h samples, the generation of epitope-related fragments in the “digestion maps” substantially corresponded with actual CTL induction (17/18 epitopes). Furthermore, this excellent prognostic accuracy indicates that the murine-i20S digestion assay can be successfully used as a substitute for the ELISPOT assay.

### Application of the murine-i20S digestion assay to predict CTL induction for the HLA-A3 supertype epitope

**Fig 3:**
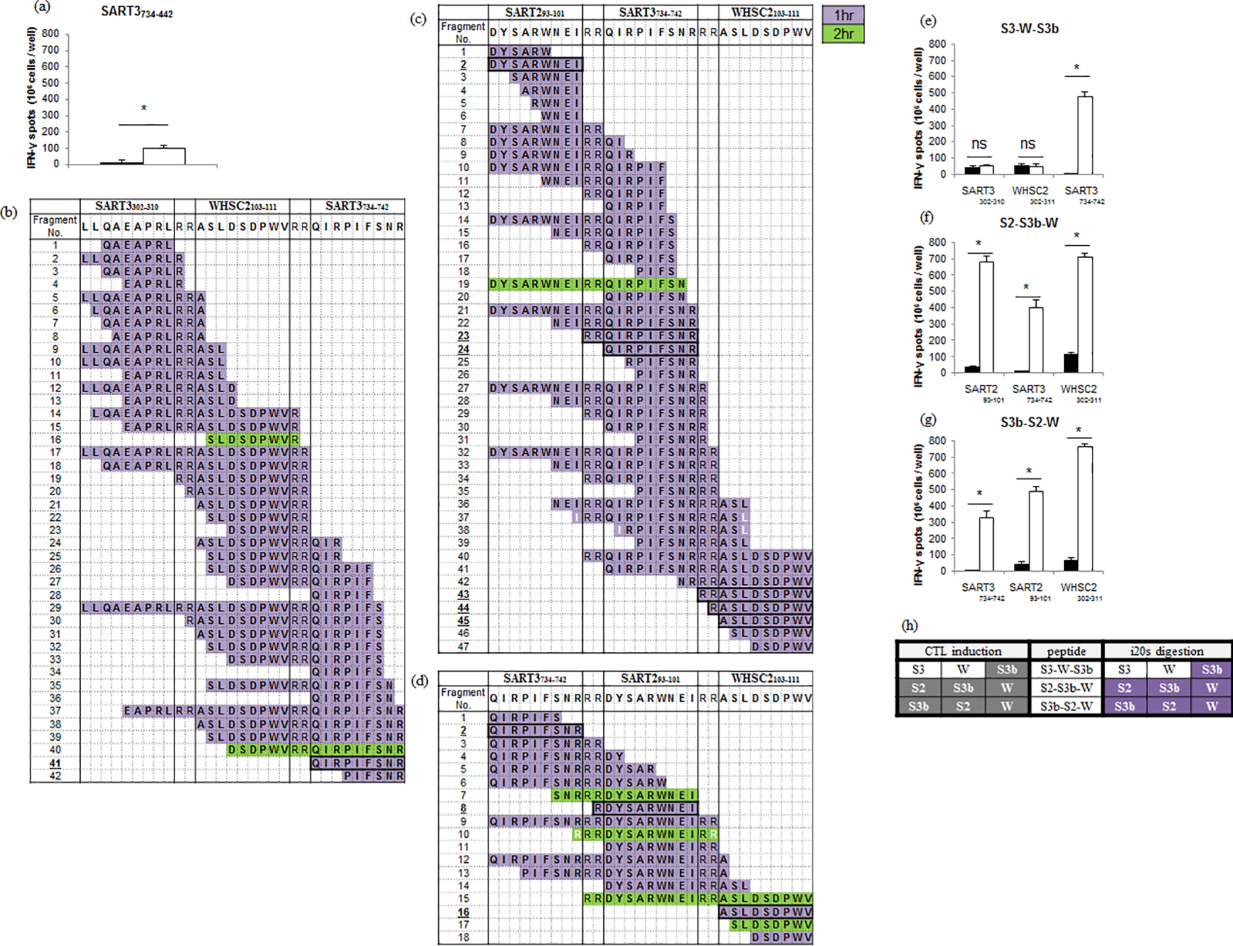
Application of the murine-i20S digestion assay to predict CTL induction for the *HLA-A3* supertype-restricted epitope. (a) IFN-γ ELISPOT assay results from SART3_734-742_ epitope-treated mice. Data represent mean ± s.d. (n = 3–4); open bars, target epitope peptide stimulation; closed bars, negative control peptide stimulation; * Student’s t-test, p < 0.05. (b)-(d) The digestion map of S3-W-S3b, S2-S3b-W, S3b-S2-W constructs from the murine-i20S digestion assay. (e)-(g) IFN-γ ELISPOT assay results from S3-W-S3b, S2-S3b-W, and S3b-S2-W-treated mice. Peptides used for in vitro re-stimulation are shown at the bottom. (h) Comparison of the results between CTL induction and the murine-i20S digestion assay.

In addition to *HLA-A2* and *A24*, worldwide frequency of *HLA-A3* supertype alleles (including *A3*, *A11*, *A31*, *A33*, and *A68*) is also high. Thus, we next investigated whether the murine-i20S digestion assay is applicable for *HLA-A3* epitopes. SART3_734-742_ is reported as an *HLA-A3* supertype -restricted epitope [41], and actually induced specific CTLs in HLA-A*3101 KI mice (Fig 3A).

In order to evaluate whether SART3_734-742_ is adequately cleaved, three trivalent SLPs in which SART3_734-742_ (S3b) is arranged at diverse positions (C-terminus, middle, N-terminus) were synthesized (Table 2) and a prospective analysis was performed with the murine-i20S digestion assay. Fig 3B–3D shows the resulting digestion map of S3-W-S3b, S3b-S2-W, and S2-S3b-W. SART3_734-742_ epitope-related fragments were detected from all three SLPs. These results indicate that the SLPs would be able to induce specific CTLs for SART3_734-742_.

**Table 2:** Sequences of trivalent SLPs including SART3_734-742_.

Name	Orientation	Epitope Sequence
S3-W-S3b	SART3_302-310_-RR-WHSC2_103-112_-RR-SART3_734-742_	**LLQAEAPRL**RR**ASLDSDPWV**RR**QIRPIFSNR**
S2-S3b-W	SART2_93-101_-RR-SART3_734-742_-RR-WHSC2_103-113_	**DYSARWNEI**RR**QIRPIFSN**RR**RASLDSDPWV**
S3b-S2-W	SART3_734-742_-RR-SART2_93-101_-RR-WHSC2_103-113_	**QIRPIFSN**RRR**DYSARWNEI**RR**ASLDSDPWV**
		*Epitope sequences are shown in bold*

Accordingly, CTL induction was confirmed using HLA-A*3101 KI mice. As a result, the specific CTLs for SART3_734-742_ were induced from all three SLPs (Fig 3E–3G). Furthermore, including the other epitopes, ELISPOT results perfectly matched predictions from the murine-i20S digestion assay (9/9 epitopes = 100%, Fig 3H). In addition to the data presented in Fig 2N, this result supports the overall accuracy of the murine-i20S digestion assay for the prediction of ELISPOT results. From the i20S digestion assay results, S2-S3b-W and S3b-S2-W were finally selected as the SLPs for which all three epitopes could induce specific CTLs”.

### Antitumor efficacy of SLP vaccines in epitope-expressing syngeneic tumors

**Fig 4:**
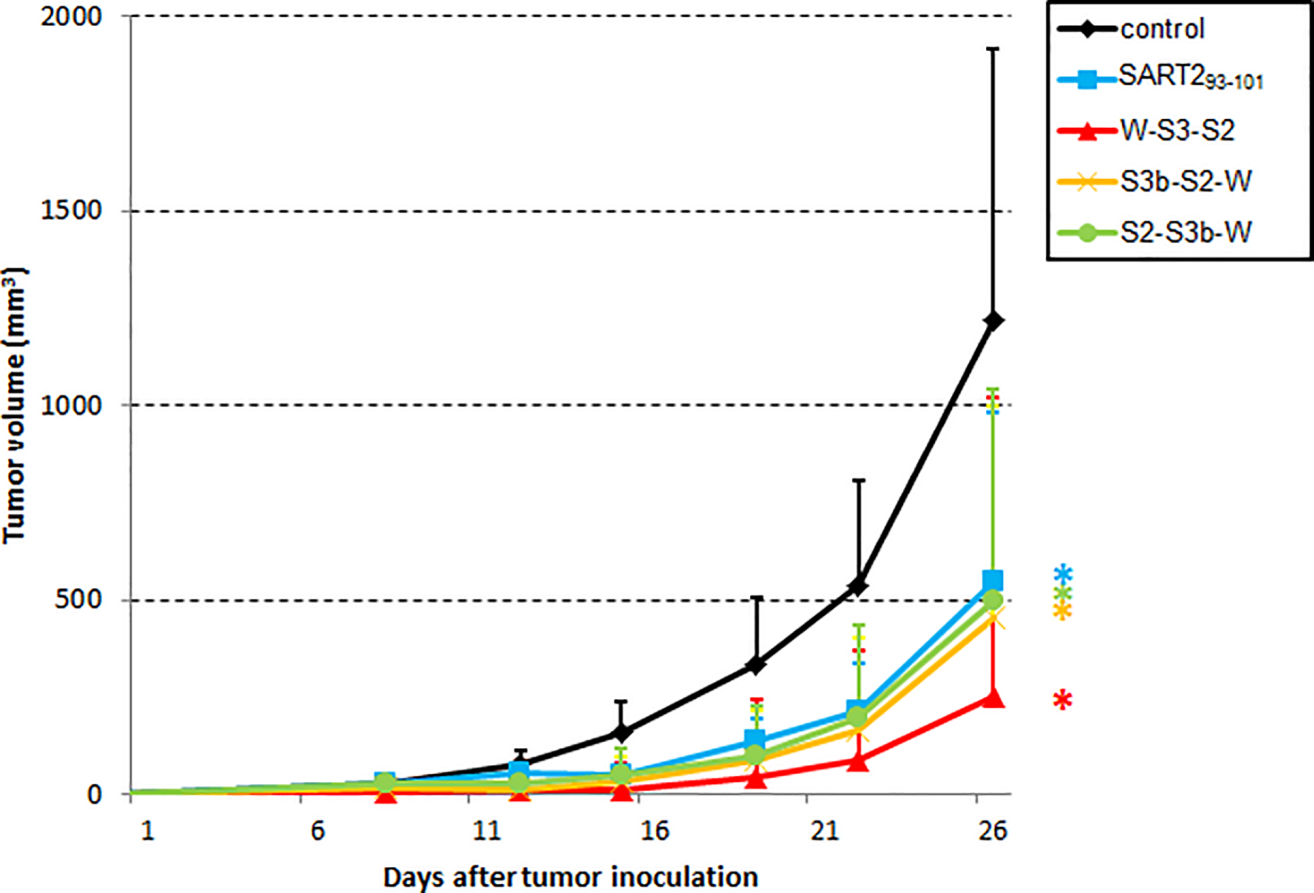
Antitumor efficacy of multivalent SLP vaccines in the syngeneic tumor mouse model. *HLA-A24* KI mice (n = 10) were subcutaneously treated with distilled water (control), SART2_93-101_ or multivalent SLPs that were emulsified with Montanide ISA-51VG. Seven days after the final vaccination, B16F10.A24/SART2_93-101_ cells (5 × 10^6^ cells) were subcutaneously engrafted into vaccinated mice. Tumor sizes were monitored with calipers, twice per week. Data are presented as mean tumor volume ± s.d.; * Student’s t-test, p < 0.05 vs. control group.

In order to evaluate the antitumor efficacy of suitably-digested trivalent SLPs, we immunized HLA-A*2402 KI mice with W-S3-S2, S3b-S2-W, and S2-S3b-W, and monitored the growth of HLA-A*2402- and SART2_93-101_-expressing B16F10 syngeneic tumors. We chose these SLPs to confirm whether the epitope position also affects antitumor efficacy (SART2_93-101_ is positioned at the C-terminus, middle and N-terminus, respectively). As shown in Fig 4, tumor sizes in the SART2_93-101_, W-S3-S2, S3b-S2-W, and S2-S3b-W-vaccinated groups were significantly lower than in control mice (p < 0.05). Furthermore, 20%, 50%, 40%, and 20% of mice in the SART2_93-101_, W-S3-S2, S3b-S2-W, and S2-S3b-W treatment groups, respectively, were tumor-free on day 26. These results demonstrate that the murine-i20S digestion assay can predict not only the induction of CTLs, but also their antitumor efficacy.

### Comparison of i20S cleavage preferences between mouse and human

**Fig 5:**
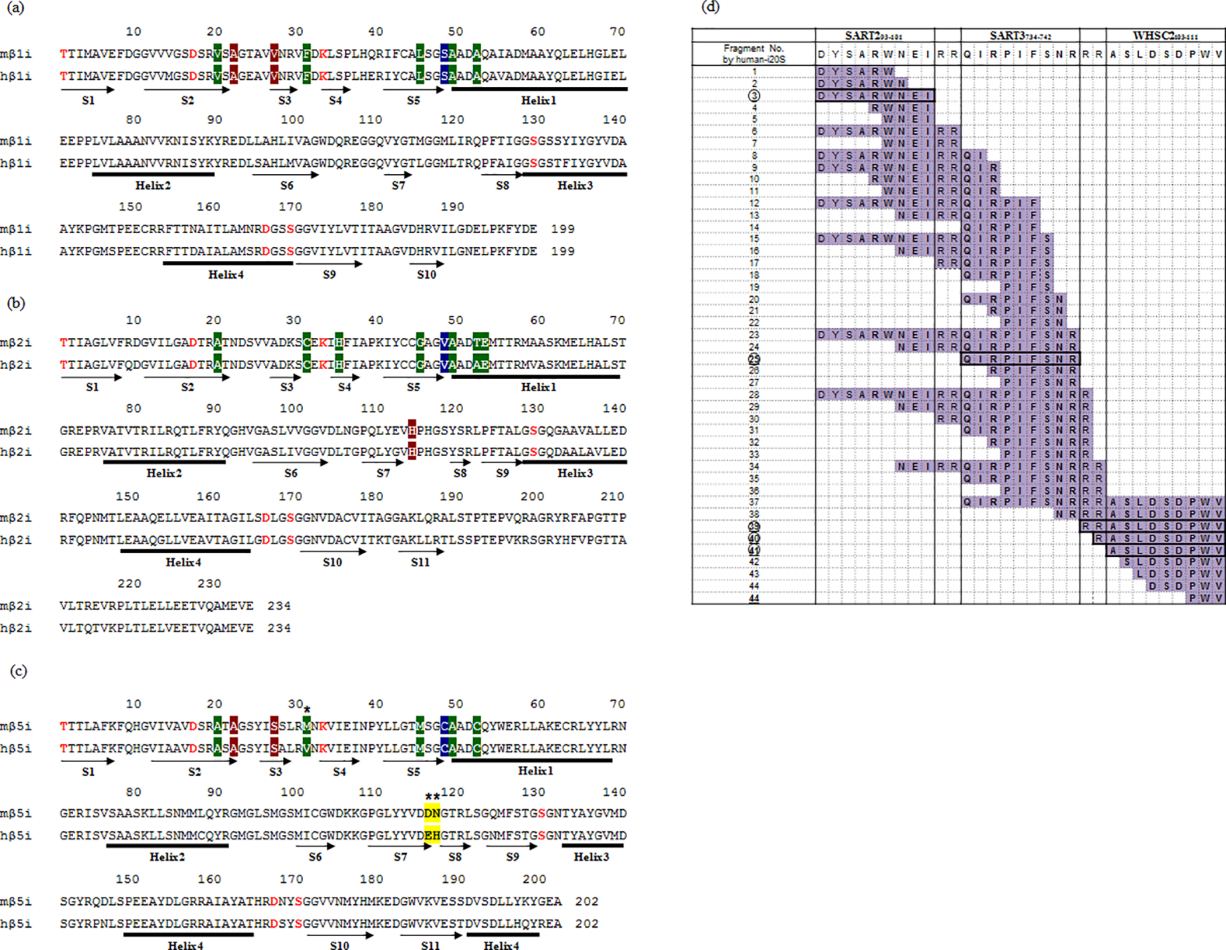
Comparison of cleavage preferences between murine-i20S and human-i20S. (a)-(c) Sequence alignments of the catalytic β-subunits from mouse-i20S and human-i20S. Important residues on the active site are displayed in red. The substrate-recognizing pocket for primed substrate was called S’, and those for unprimed substrate were called S1, S2, and S3 respectively, from the C-terminal anchor residue of the epitope. Residues contributing to substrate-specificity pockets are highlighted by colored boxes: S1 pocket, green; S2 pocket, blue; S3 pocket, brown; S’ pocket, yellow. Different residues between murine and human β-subunits are marked with asterisks. Secondary structures (S: β-sheet & Helix) are indicated under the sequence. (d) The digestion map of S2-S3b-W in the human-i20S digestion assay. The numbers of epitope-related fragments detected in the murine-i20S digestion assay (Fig 3F) are enclosed in circles.

Finally, we compared the murine-i20S and human-i20S digestion assays to evaluate whether this approach is suitable for the design of human multivalent SLP vaccines. Amino-acid sequence comparison between the active center of the three catalytic subunits in murine i20S and human i20S revealed that the residues of the active sites of β1i, β2i, and β5i and the substrate-specificity pockets of β1i and β2i were completely consistent [42] (Fig 5A and 5B & 5C). In addition, the residues in the substrate binding pocket which recognizes the C-terminal anchor residue of an epitope (S1) and the next residue (S’) had similar amino acids. Therefore, the digestion pattern of human i20S was predicted to be similar to that of murine i20S. Thus, we compared the cleavage patterns from the human-i20S and murine-i20S digestion assays using the S2-S3b-W construct (Fig 5D). As for the murine-i20S digestion result (Fig 3F), epitope-related fragments of all three epitopes could be detected using the human assay. These results indicate that the cleavage pattern of human-i20S is very similar to murine-i20S, especially in regard to epitope-related fragments, and that the human-i20S digestion assay would be suitable to predict human CTL induction.

## Discussion

In this study, we established a murine-i20S digestion assay and confirmed its validity for the prediction of CTL induction. Murine-i20S digestion patterns were shown to predict CTL induction responses to a multivalent SLP vaccine with excellent accuracy. In addition, we have established a novel syngeneic tumor mouse model which can be used to evaluate the antitumor efficacy of SLP-vaccines that include the SART2_93-101_ epitope. To date, there are few animal models that have been shown to be suitable for evaluating cancer vaccine efficacies via HLA. Together with the i20S digestion assay, this syngeneic mouse model represents a powerful tool for the development of novel immunotherapy approaches. Using this model, we have thus demonstrated that the murine-i20S digestion assay may be a viable substitute for more complex and costly studies using HLA-expressing mice.

In order to set judgment criteria for suitable epitope cleavage, we focused on “epitope-related fragments” since i20S contributes mainly to determine the C-terminus of epitope peptides. Previous work has shown that N-terminus-extended epitope peptides are trimmed by amino-exopeptidases to become intact epitopes [29, 30]. Compared to ELISPOT data, we found that the number of epitope-related fragments generated appeared to be related to the CTL response level. For example, WHSC2_103-111_-specific CTL induction of S2-S3-W was stronger than that of S3-S2-W (Fig 2L & 2H, respectively), while S2-S3-W was superior to S3-S2-W also in numbers of epitope-related fragments (Fig 2F & 2B, respectively). A similar tendency was also seen for SART3_302-310_ between S2-W-S3 and W-S2-S3 (Fig 2C, 2E & 2I and 2K, respectively). Moreover, in the case of SART2_93-101_ of W-S3-S2, S3b-S2-W, and S2-S3b-W, W-S3-S2 which generated three epitope-related fragments showed both the highest CTL response and tumor-free ratio (Figs 3B–3G & 4).

A total of nine trivalent SLPs were synthesized for evaluation in the murine-i20s digestion assay. Across two different comparison studies, we found that ELISPOT assay results were predicted by the murine-i20S digestion assay for 25/27 epitopes (92.6%), when fragments were analyzed regardless of incubation time. Although there are some limitations of this assay, we concluded that the murine-i20S digestion assay can predict CTL induction and antitumor effects using HLA-expressing mice. Based on our prospective analysis for SART3_734-742_, it was indicated that SART3_734-742_-specific CTLs can be induced from all three trivalent SLPs that we tested.

To predict potential T-cell epitopes from whole protein sequences, there are several computer programs available [43–48]. Some of these programs are based on the results of actual i20S digestion patterns, however, the accuracy of these programs is not high because the i20S cleavage pattern is too complicated to be predicted [49]. In this study, we have shown that the in vitro murine-i20S digestion assay can achieve an accurate prediction of CTL induction and antitumor effects. There are three requirements that must be satisfied for proper epitope presentation: (i) suitably digested by the proteasome (ii) transported to the endoplasmic reticulum by the transporter associated with antigen processing (TAP) (iii) bound to MHC class I. The epitopes used in this study have already been confirmed to work in human, thereby, satisfying (ii) and (iii). Thus, CTL induction is determined only by whether SLP is suitably digested. In this manner, the murine-i20S digestion assay is a useful tool for multivalent SLP vaccine screening. We consider that such an experimental assay is absolutely necessary for epitope prediction. Further work is needed to evaluate whether the murine-i20S digestion assay is suitable to predict T-cell epitopes from whole protein sequences.

We also compared CTL induction between SLPs and single epitope peptides, as previously investigated. In most cases, our data also indicated superior CTL induction of SLPs compared to single epitope peptides if only they were induced. The results from trivalent SLP screening revealed that the position of each epitope peptide is important for the design of multivalent SLP vaccines. For example, WHSC2_103-111_ and SART3_302-310_ induced specific CTLs only when they were positioned at the C-terminus of a trivalent SLP (Figs 2H, 2I, 2K, 2L, 3F & 3G). Previous work has shown that residues on both sides of the C-terminal anchor site influence proteasomal cleavage [50, 51]. An Arg-linker was expected to provide preferential cleavage sites, however, in the case of WHSC2_103-111_ and SART3_302-310_, C-terminal Arg residues did not promote efficient cleavage. Since a universal linker has not yet been found, we developed the murine-i20S digestion assay to determine optimal peptide design. When these epitopes were positioned at the C-terminus of a multivalent SLP vaccine while constantly exposing their C-terminus, they were precisely cleaved into intact epitopes by amino-exopeptidases. However, we also identified epitopes that induced specific CTLs regardless of their position, such as SART2_93-101_ and SART3_734-742_. These position-independent epitopes are thus important for the design of multivalent SLP vaccines. The murine-i20S digestion assay makes it significantly easier to identify the position-dependency of component epitopes in vitro.

Comparison of the amino-acid sequences of the murine- and human-i20S subunits revealed that the residues of the active site and the substrate-specificity pockets of β1i and β2i matched perfectly (Fig 5A & 5B). A previous report [42] showed that β1i preferentially cleaves after small, hydrophobic, and branched residues, such as isoleucine (Ile) or leucine (Leu) (carried by WHSC2_103-111_ and SART2_93-101_), and that β2i cleaves after basic and neutral hydrophilic residues, like Arg (carried by SART3_734-742_). In addition, PR-957, which is known as a β5i-specific inhibitor, inhibited murine-i20S as well as human-i20S, indicating that they have similar substrate cleavage properties despite the D115E and N116H substitutions that are found within β5i [42]. Such observations support the similar digestion patterns between murine- and human-i20S that we determined experimentally (Fig 5D).

In summary, our results demonstrate that the i20S digestion assay is a useful strategy for determining the optimal design of multivalent SLP vaccines. Vaccines designed using the murine-i20S digestion assay demonstrated in vivo antitumor efficacy and optimal induction of specific CTLs from all epitopes, and may thus be novel candidates for use in treating human cancer. To make even longer vaccines, we need further information regarding the position-dependency of each epitope, and this can be obtained using the i20S digestion assay. We believe that in vitro-designed multivalent SLP vaccines are an important innovation in the development of therapeutic strategies for tumors and infectious disease.

## Supporting information

S1 Checklist NC3Rs ARRIVE guidelines checklist. (PDF)

## Abbreviations

SLP: synthetic long peptide
CTL: cytotoxic T lymphocyte
IFN-μΛ: interferon-gamma
PBMC: peripheral blood mononuclear cell
HLA: human leukocyte antigen
i20S: 20S immunoproteasome
HPLC: high performance liquid chromatography
LC-MS/MS: liquid chromatography-tandem mass spectrometry
KI: knock-in
Tg: transgenic.
