# Correction: Development of a novel immunoproteasome digestion assay for synthetic long peptide vaccine design

**DOI:** 10.1371/journal.pone.0205567

**Published:** 2018-10-04

**Authors:** Hiroshi Wada, Atsushi Shimizu, Toshihiro Osada, Yuki Tanaka, Satoshi Fukaya, Eiji Sasaki

There are typographical errors in the fourth and fifth sentences of the Abstract. “IFN-μ” should appear as “IFN-γ.” The correct sentences are: However, to confirm whether a multivalent vaccine can induce an individual epitope-specific CTL, the only viable screening strategies currently available are interferon-gamma (IFN-γ enzyme-linked immunospot (ELISPOT) assays using human peripheral blood mononuclear cells, or expensive human leukocyte antigen (HLA)-expressing mice. In this report, we evaluated the use of our developed murine-20S immunoproteasome (i20S) digestion assay and found that it could predict the results of IFN-γ ELISPOT assays.

There is a typographical error in the Abbreviations. “IFN-μΛ, interferon-gamma” should appear as “IFN-γ, interferon-gamma.”
